# Tryptophan Ameliorates Metabolic Syndrome by Inhibiting Intestinal Farnesoid X Receptor Signaling: The Role of Gut Microbiota–Bile Acid Crosstalk

**DOI:** 10.34133/research.0515

**Published:** 2024-12-13

**Authors:** Jiayi Chen, Hao Yang, Yingjie Qin, Xinbo Zhou, Qingquan Ma

**Affiliations:** College of Animal Science and Technology, Northeast Agricultural University, Harbin 150030, China.

## Abstract

**Background and Aims:** Metabolic syndrome (MS) is a progressive metabolic disease characterized by obesity and multiple metabolic disorders. Tryptophan (Trp) is an essential amino acid, and its metabolism is linked to numerous physiological functions and diseases. However, the mechanisms by which Trp affects MS are not fully understood.

**Methods and Results:** In this study, experiments involving a high-fat diet (HFD) and fecal microbiota transplantation (FMT) were conducted to investigate the role of Trp in regulating metabolic disorders. In a mouse model, Trp supplementation inhibited intestinal farnesoid X receptor (FXR) signaling and promoted hepatic bile acid (BA) synthesis and excretion, accompanied by elevated levels of conjugated BAs and the ratio of non-12-OH to 12-OH BAs in hepatic and fecal BA profiles. As Trp alters the gut microbiota and the abundance of bile salt hydrolase (BSH)-enriched microbes, we collected fresh feces from Trp-supplemented mice and performed FMT and sterile fecal filtrate (SFF) inoculations in HFD-treated mice. FMT and SFF not only displayed lipid-lowering properties but also inhibited intestinal FXR signaling and increased hepatic BA synthesis. This suggests that the gut microbiota play a beneficial role in improving BA metabolism through Trp. Furthermore, fexaramine (a gut-specific FXR agonist) reversed the therapeutic effects of Trp, suggesting that Trp acts through the FXR signaling pathway. Finally, validation in a finishing pig model revealed that Trp improved lipid metabolism, enlarged the hepatic BA pool, and altered numerous glycerophospholipid molecules in the hepatic lipid profile.

**Conclusion:** Our studies suggest that Trp inhibits intestinal FXR signaling mediated by the gut microbiota–BA crosstalk, which in turn promotes hepatic BA synthesis, thereby ameliorating MS.

## Introduction

Metabolic syndrome (MS) is a metabolic disorder characterized by obesity, hyperglycemia, hyperlipidemia, and hypertension. MS, which is strongly associated with cardiovascular disease, metabolic dysfunction-associated steatotic liver disease (MASLD), and type 2 diabetes, has become a major public health threat, necessitating urgent improvements in the management of metabolic disorders [1,2]. Bile acids (BAs) play a pivotal role in the improvement of MS. BAs are synthesized in hepatocytes and are essential components of bile, contributing to the absorption of fats and fat-soluble vitamins, and regulating cholesterol levels. Primary BAs synthesized in hepatocytes are excreted into the intestines via gallbladder contraction after food intake. The BAs entering the intestines emulsify fats, facilitating the digestion and absorption of fat-soluble substances. Most primary BAs are actively reabsorbed as conjugated BAs at the terminal ileum, while a small amount of BAs is deconjugated by bile salt hydrolase (BSH) produced by the gut microbiota into unconjugated BAs, which are then dehydroxylated to secondary BAs. Most secondary BAs are reabsorbed in the ileum and returned to the liver via the portal vein system [3,4]. The gut microbiota can metabolize BAs and influence BA signaling, while BAs can, in turn, influence the composition of the gut microbiota [5]. Recent studies have demonstrated that the interaction between BAs and the gut microbiota is closely related to metabolic diseases [6,7]. The gut microbiota–BA crosstalk may be involved in the pathogenesis of MS and may accelerate its progression.

BAs are also important signaling molecules that play a role in various metabolic processes through direct or indirect activation of the nuclear receptor farnesoid X receptor (FXR) [8]. Given its pivotal role in the metabolism of BAs, lipids, and glucose, FXR represents a promising drug target for the treatment of metabolic and hepatic diseases [9]. Hepatic FXR activation inhibits the expression of cholesterol 7α-hydroxylase (CYP7A1), the rate-limiting enzyme for the conversion of cholesterol to BAs, via the short heterodimer partner (SHP). Intestinal FXR activation induces increased production of fibroblast growth factor-15 (FGF15) [FGF15 is the mouse homolog of FGF19 (FGF19 in humans and pigs), and although the amino acid residues are different, they have similar functions], which is subsequently secreted into the portal vein and circulated to the liver, inhibiting hepatic BA biosynthesis by binding to fibroblast growth factor receptor 4 (FGFR4) [10,11]. Increasing evidence suggests that inhibition of the intestinal FXR signaling pathway induces many positive effects, such as the amelioration of obesity, hyperlipidemia, insulin resistance, and MASLD [12–15].

Tryptophan (Trp), one of the aromatic amino acids that cannot be synthesized by the body, is an essential component of the human diet. Trp metabolism regulates a wide range of physiological and pathological processes, such as growth regulation, metabolism, mood, and immune responses. Trp exerts its physiological effects through its metabolites. Trp metabolism is divided into 2 major components: host-metabolized and microbially mediated Trp metabolism. Trp metabolites produced by the gut microbiota are important signaling molecules for microbial communities and host communication, contributing to the maintenance of intestinal and systemic homeostasis [16,17]. Oral Trp supplementation has been shown to attenuate MASLD in mice [18]. The lower the level of indole produced by microbial metabolism of Trp, the higher the fat deposition in the liver [19]. Trp also prevents metabolic damage by reducing levels of inflammatory factors [20]. Therefore, Trp may be a potential nutrient for preventing and ameliorating MS. In previous studies, we supplemented the chow diet (CD) of mice with 3 aromatic amino acids, including Trp. Comprehensive analysis of the transcriptome and metabolome indicated that these amino acids increased hepatic BA synthesis [21]. Therefore, we speculate that Trp may have a important effect on regulating BA metabolism.

In the present study, Trp altered the microbiota composition and BA profile. It was found to inhibit the intestinal FXR signaling pathway and increase hepatic BA synthesis in both mice and finishing pigs. These findings indicate a mechanistic relationship between Trp and the gut microbiota–BA crosstalk in alleviating MS.

## Materials and Methods

### Animal study

All animal procedures were conducted in strict accordance with the Guidelines for the Care and Use of Laboratory Animals of Northeast Agricultural University (NEAU-[2011]-9), and the experimental protocols were approved by the University Animal Care Committee. Trp intervention in a mouse model: Male C57BL/6J mice were obtained from HFK Co. Ltd. (SPF, Beijing, China) at 5 weeks of age. Mice were housed under specific pathogen-free (SPF) conditions in plastic rodent cages at 22 to 24 °C with a 12-h light/dark cycle and had ad libitum access to food and water throughout the experimental period. The diets consisted of a normal CD (10% fat; 3,660 kcal/g; Xietong Biotechnology Company, Nanjing, China) and a high-fat diet (HFD) (60% fat; 5,128 kcal/g; Xietong Biotechnology Company, Nanjing, China). Mice were acclimated to the normal CD for 1 week before the experiments began. Each group had 8 replicates. Body weight and food consumption were monitored weekly. At the end of the experiment, all mice were euthanized for tissue collection.

**Fig. 1.**
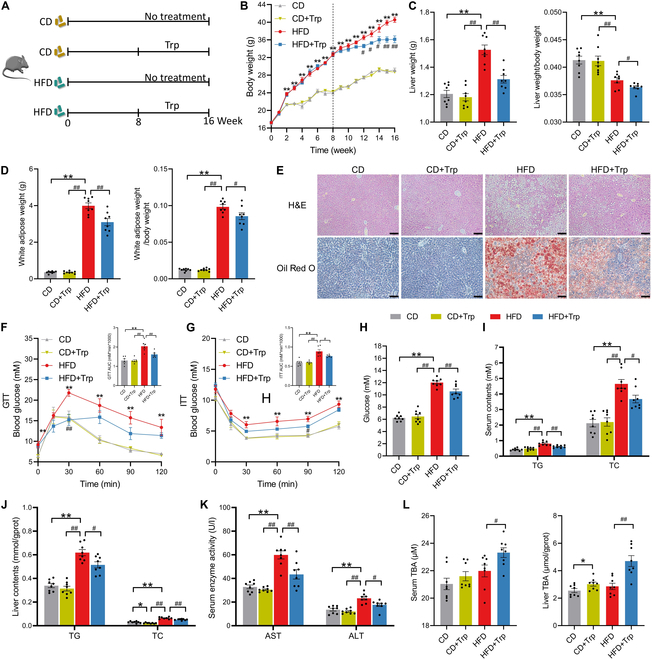
Trp ameliorates HFD-induced MS in mice. (A) Schematic of the experimental procedure to test the role of Trp in MS. (B) Changes in body weight. (C) Liver weight, liver weight-to-body weight ratio. (D) WAT and WAT weight-to-body weight ratio. (E) H&E and Oil Red O staining images of liver. Scale bar, 50 μm. (F and G) GTT, AUC of GTT, ITT, and AUC of ITT. (H) Serum glucose level. (I and J) TC and TG levels in serum and liver. (K) Serum AST and ALT levels. (L) Serum and liver TBA levels. Values are means ± SEM (*n* = 8 per group). For statistical analysis, a 2-tailed unpaired Student’s *t* test was used. *P < 0.05, **P < 0.01 versus the CD group. ^#^*P* < 0.05, ^##^*P* < 0.01 versus the HFD group.

In the treatment study, mice were weighed and randomly assigned to 2 diets. To induce obesity, mice were fed the HFD diet, while the CD diet served as the control. Obesity was considered established when the body weight of the HFD-fed mice was 20% higher than that of the CD-fed mice. After 8 weeks of HFD feeding to induce obesity, Trp was added to both diets at twice the original molar concentration and continued for another 8 weeks. Mice fed CD and HFD without Trp served as controls (Fig. 1A). The actual concentration of Trp in each group is as follows: CD group, 0.20%; CD + Trp group, 0.40%; HFD group, 0.27%; and HFD + Trp group, 0.54%. To ensure isonitrogenous diets and minimize the impact of other amino acids, additional nitrogen in the CD and HFD groups was compensated by supplementing with an amino acid mixture excluding Trp, added in proportion.

It is important to note that during the final week of the intervention, fresh fecal samples (gut microbiota with or without its metabolites) from the HFD + Trp group were aseptically collected for subsequent fecal microbiota transplantation (FMT) or sterile fecal filtrate (SFF) preparations. FMT and SFF were prepared as described in [21]. After feeding HFD for 8 weeks, mice underwent gavage with a mixed antibiotic solution (1 g/l neomycin sulfate, 1 g/l metronidazole, 1 g/l ampicillin, and 0.5 g/l vancomycin, 10 ml/kg) for 1 week. Subsequently, mice were orally gavaged with FMT (10 ml/kg/day) or SFF (10 ml/kg/day), while the control group received sterile saline orally (10 ml/kg/day). Gavage continued for 5 weeks with HFD maintained throughout (Fig. 3E).

In the FXR regulation study, mice were divided into 4 groups after 8 weeks on an HFD: (a) HFD vehicle (HFD), (b) HFD supplemented with 100 mg/kg/day Gly-MCA by gavage (HFD + Gly-MCA), (c) HFD vehicle supplemented with additional Trp (HFD + Trp), and (d) HFD supplemented with additional Trp and 100 mg/kg/day fexaramine by gavage (HFD + Trp + Fex). The intervention lasted 6 weeks (Fig. 4A). The actual concentration of Trp in each group is as follows: HFD group and HFD + Gly-MCA, 0.27%; HFD + Trp group and HFD + Trp + Fex, 0.54%.

Trp intervention in a finishing pig model: We purchased 12 healthy male finishing pigs (Large White) from a breeding pig farm (Jubao, Suihua City, Heilongjiang Province, China). A total of 12 finishing pigs were randomly allocated to 2 dietary treatments: a control group fed the basal diet, and a Trp group fed the basal diet supplemented with 0.78% Trp. The actual concentration of Trp in each group is as follows: control group, 0.14%; Trp group, 0.92%. To ensure isonitrogenous diets and minimize the effects of other amino acids, additional nitrogen in the control group was compensated by supplementing with an amino acid mixture excluding Trp, added proportionally. There were 6 replicates per group, with 1 pig per replicate. Pigs were individually housed in separate pens within the same room. The duration of the experiment was 30 days (Fig. 5A). Trp was purchased from Hebei Huayang Biological Technology Co. Ltd. (≥99.0% purity, Hengshui, China).

### 16S rDNA amplicon sequencing

Polymerase chain reaction (PCR) amplification was performed using the following primers: 341F (CCTAYGGGRBGCASCAG) and 806R (GGACTACNNGGGTATCTAAT). All PCRs were carried out with Phusion High-Fidelity PCR Master Mix (New England Biolabs). The same volume of 1× loading buffer (containing SYB green) was mixed with PCR products, and electrophoresis was operated on 2% agarose gel for detection. Samples with bright main strip between 400 and 450 base pairs (bp) were chosen for further experiments. PCR products were mixed in equidensity ratios. Then, mixture PCR products were purified with Qiagen Gel Extraction Kit (Qiagen, Germany). Sequencing libraries were generated using TruSeq DNA PCR-Free Sample Preparation Kit (Illumina, USA). The library quality was assessed on the Qubit 2.0 Fluorometer (Thermo Fisher Scientific) and Agilent Bioanalyzer 2100 system. At last, the library was sequenced on an Illumina HiSeq 2500 platform and 250-bp paired-end reads were generated [22].

### Microbiota data analysis

Sequences analysis was performed by Uparse software (Uparse v7.0.1001). Sequences with ≥97% similarity were assigned to the same operational taxonomic units (OTUs). Representative sequence for each OTU was screened for further annotation. For each representative sequence, the GreenGene Database was used based on RDP classifier (version 2.2) algorithm to annotate taxonomic information. Multiple sequence alignment was conducted using the MUSCLE software. OTU abundance information was normalized using a standard of sequence number corresponding to the sample with the least sequences. Subsequent analysis of alpha diversity and beta diversity was performed based on the output normalized data. Alpha diversity was applied to analyze the complexity of species diversity for a sample. Beta diversity analysis was used to evaluate differences of samples in species complexity. Beta diversity on both weighted and unweighted UniFrac was calculated by QIIME software (version 1.7.0). Principal coordinate analysis (PCoA) was performed to get principal coordinates and visualize from complex, multidimensional data. PCoA analysis was displayed in R software (version 2.15.3). LEfSe (LDA effect size) analyses were performed using the Kruskal–Wallis rank sum test for all characterized species, and significantly different species were obtained by detecting the difference in species abundance between different groups. The Wilcoxon rank sum test was then used to test whether all subspecies of the significantly different species obtained in the previous step converged to the same taxonomic level. Finally, linear discriminant analyses (LDAs) were used to obtain the final divergent species [23]. In addition, Spearman correlation between the BA profile and significantly different microbes and radar plots of the relative abundance of BSH-enriched microbes were produced using the ChiPlot website.

### Statistical analysis

All data are presented as mean ± standard error of the mean (SEM). Statistical analysis was conducted using a 2-tailed unpaired Student’s *t* test in SPSS Statistics (Chicago, USA). A significance level of *P* < 0.05 was adopted. The statistical significance is denoted as follows: *P < 0.05; **P < 0.01; ^#^*P* < 0.05, ^##^*P* < 0.01.

Other methods are in the Supplementary Materials.

## Results

### Trp ameliorates HFD-induced MS and is associated with enhanced BA metabolism in mice

To investigate the regulatory effects of Trp on MS, mice were fed an HFD for 8 weeks to induce MS, followed by 8 weeks of Trp intervention. The effect of Trp on a normal CD-fed mice was also examined (Fig. 1A). Compared to the CD group, the HFD group exhibited lower food intake, higher energy intake, and increased weight gain in mice (*P* < 0.05). Following Trp intervention, Trp treatment in the HFD group reduced weight gain (*P* < 0.05), while Trp treatment in the CD group had no effect on weight gain (Fig. 1B and Fig. S1A and B). The liver index and white adipose tissue (WAT) index also increased in the HFD group compared to the CD group (*P* < 0.05). Hematoxylin and eosin (H&E) and Oil Red O staining revealed larger lipid droplets and increased ballooning degeneration in the hepatocytes of HFD-treated mice. However, under HFD conditions, Trp intervention led to a reduction in WAT and remission of hepatic steatosis (*P* < 0.05) (Fig. 1C to E).

In order to investigate the impact of Trp supplementation on glucose homeostasis, we conducted glucose tolerance tests (GTTs) and insulin tolerance tests (ITTs). The results demonstrated that an HFD resulted in an increase in the area under the curve (AUC) of GTT and ITT assay, as well as an elevation in serum glucose levels. However, under HFD conditions, Trp intervention led to a reduction in AUC of the GTT and ITT assay and serum glucose levels (*P* < 0.05), indicating that Trp significantly improved glucose tolerance (Fig. 1F to H). The HFD also significantly increased serum and liver levels of triglycerides (TGs) and total cholesterol (TC), as well as serum aspartate aminotransferase (AST) and alanine aminotransferase (ALT). Trp alleviated HFD-induced hyperlipidemia, as evidenced by reduced TG and TC levels in serum and liver (*P* < 0.05). Trp significantly reduced serum AST and ALT levels under MS conditions, indicating that liver damage was reduced by Trp (*P* < 0.05) (Fig. 1J and K).

However, under CD conditions, Trp supplementation had no significant effect on liver weight, WAT weight, blood glucose levels, serum and liver lipid levels, and serum AST and ALT levels (Fig. 1C to K). Under MS conditions, Trp increased serum total bile acid (TBA) levels (*P* < 0.05). Interestingly, Trp increased liver TBA levels in mice with or without MS (*P* < 0.05) (Fig. 1I and L). These data suggest that Trp has no significant influence on lipid metabolism under normal metabolic conditions but can improve lipid metabolism to alleviate MS under MS conditions.

**Fig. 2.**
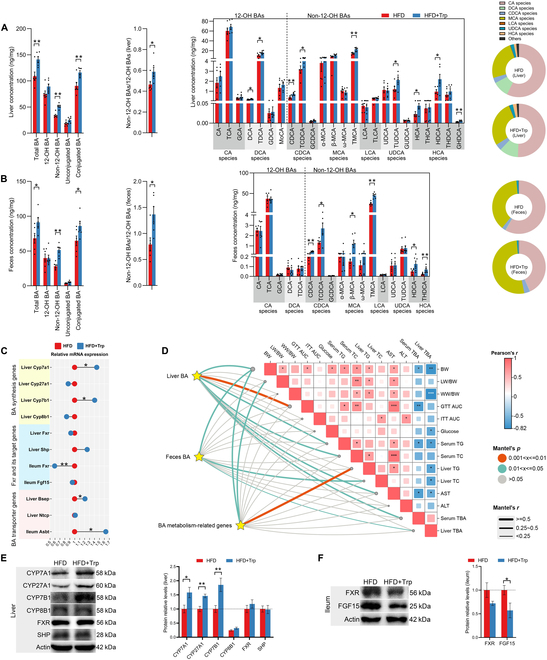
Trp alters BA metabolism. (A) Liver BA profile of mice. (B) Fecal BA profile of mice. (C) mRNA levels of BA metabolism-related genes (*Cyp7a1*, *Cyp27a1*, *Cyp7b1*, *Cyp8b1*, *Fxr*, *Shp*, *Fgf15*, *Bsep*, *Ntcp*, and *Asbt*). (D) Pairwise comparisons of indicators reflecting the degree of MS, with a color gradient denoting Pearson’s correlation coefficients. Liver BA composition, feces BA composition, and BA metabolism-related genes were related to each indicator by Mantel tests. Edge width corresponds to the Mantel’s *r* statistic for the corresponding distance correlations, and edge color denotes the statistical significance. Plotted with ChiPlot. (E) Protein expression of BA metabolism-related molecules (CYP7A1, CYP27A1, CYP7B1, CYP8B1, FXR, and SHP) in the liver. (F) Protein expression of FXR and FGF15 in the ileum. Values are means ± SEM (*n* = 6 per group). For statistical analysis, a 2-tailed unpaired Student’s *t* test was used. *P < 0.05, **P < 0.01 versus the HFD group. Ntcp, sodium taurocholate cotransporting polypeptide.

Our preliminary experiments indicated that dietary supplementation with aromatic amino acids resulted in a reduction of TG and an alleviation of hepatic steatosis, which was accompanied by an increase in BA synthesis in mice [21]. In the present study, we investigated the role of Trp, a member of the aromatic amino acids, in the BA cycle under MS conditions. The results showed that both the BAs synthesized in the liver and those excreted in the feces were altered by Trp supplementation. Trp markedly elevated the levels of numerous BA components in liver tissue, particularly non-12α-hydroxylated BAs (non-12-OH BAs), including chenodeoxycholic acid (CDCA), taurochenodeoxycholic acid (TCDCA), tauromuricholic acid (TMCA), tauroursodeoxycholic acid (TUDCA), hyocholic acid (HCA), hyodeoxycholic acid (HDCA), and glycohyodeoxycholic acid (GHDCA). In addition, the levels of deoxycholic acid (DCA) and taurodeoxycholic acid (TDCA) in 12-OH BAs were also enhanced. In fecal tissue, Trp also significantly elevated the levels of many non-12-OH BAs, including CDCA, TCDCA, β-muricholic acid (β-MCA), TMCA, HDCA, and taurohyodeoxycholic acid (THDCA). Overall, Trp increased not only TBA but also total non-12-OH and conjugated BAs in the liver and feces. It also increased the ratio of non-12-OH to 12-OH BAs in the liver and feces (*P* < 0.05). Notably, in terms of the percentage of BAs of each species, the pie charts show an increase in the proportion of MCA species and a decrease in the proportion of cholic acid (CA) species in the liver and feces of Trp-treated mice (Fig. 2A and B).

We next determined the mRNA expression levels of genes associated with BA metabolism. Consistent with elevated BA levels, the mRNA expression levels of BA synthesis genes *Cyp7a1* and oxysterol 7α-hydroxylase (*Cyp7b1*) were significantly increased by Trp supplementation. The mRNA expression level of the hepatic BA receptor *Fxr* was unchanged, but the mRNA expression level of ileum *Fxr* was markedly suppressed. In addition, the mRNA expression levels of BA transport genes, hepatic bile salt export pump (*Bsep*) and ileum apical sodium-dependent bile acid transporter (*Abst*), were also significantly increased (*P* < 0.05) (Fig. 2C). Therefore, to determine whether BA metabolism plays a key role in MS, we correlated liver BA profiles, fecal BA profiles, and BA metabolism-related genes with MS indicators using Mantel tests. Overall, liver BA profiles were significantly correlated with body weight (BW), GTT AUC, serum TG, serum TC, AST, and serum TBA; fecal BA profiles were significantly correlated with BW, liver TC, and liver TBA; and BA metabolism-related genes were significantly correlated with BW, liver weight (LW)/BW, and liver TG (*P* < 0.05) (Fig. 2D). Therefore, the BA pathway was associated with BW, lipid levels, glucose metabolism, and the degree of hepatic steatosis in mice under MS conditions. Further protein level measurements showed that the levels of proteins involved in BA synthesis (CYP7A1, CYP27A1, and CYP7B1) were markedly elevated by Trp supplementation, whereas sterol 12α-hydroxylase (CYP8B1) exhibited no notable change. No significant alteration in protein levels was observed for FXR and its target gene SHP in the liver. Although ileum FXR protein expression was unchanged, protein expression of FGF15, a downstream molecule of FXR, was decreased, indicating that intestinal FXR–FGF15 signaling was inhibited (*P* < 0.05) (Fig. 2E and F). The protein expression of liver FGF15 was also decreased (*P* < 0.05) (Fig. S2A), thereby enhancing de novo BA synthesis.

Collectively, these results suggest that Trp ameliorates HFD-induced MS and is associated with enhanced BA metabolism in mice.

### Trp ameliorates MS depending on gut microbiota–BA crosstalk

**Fig. 3.**
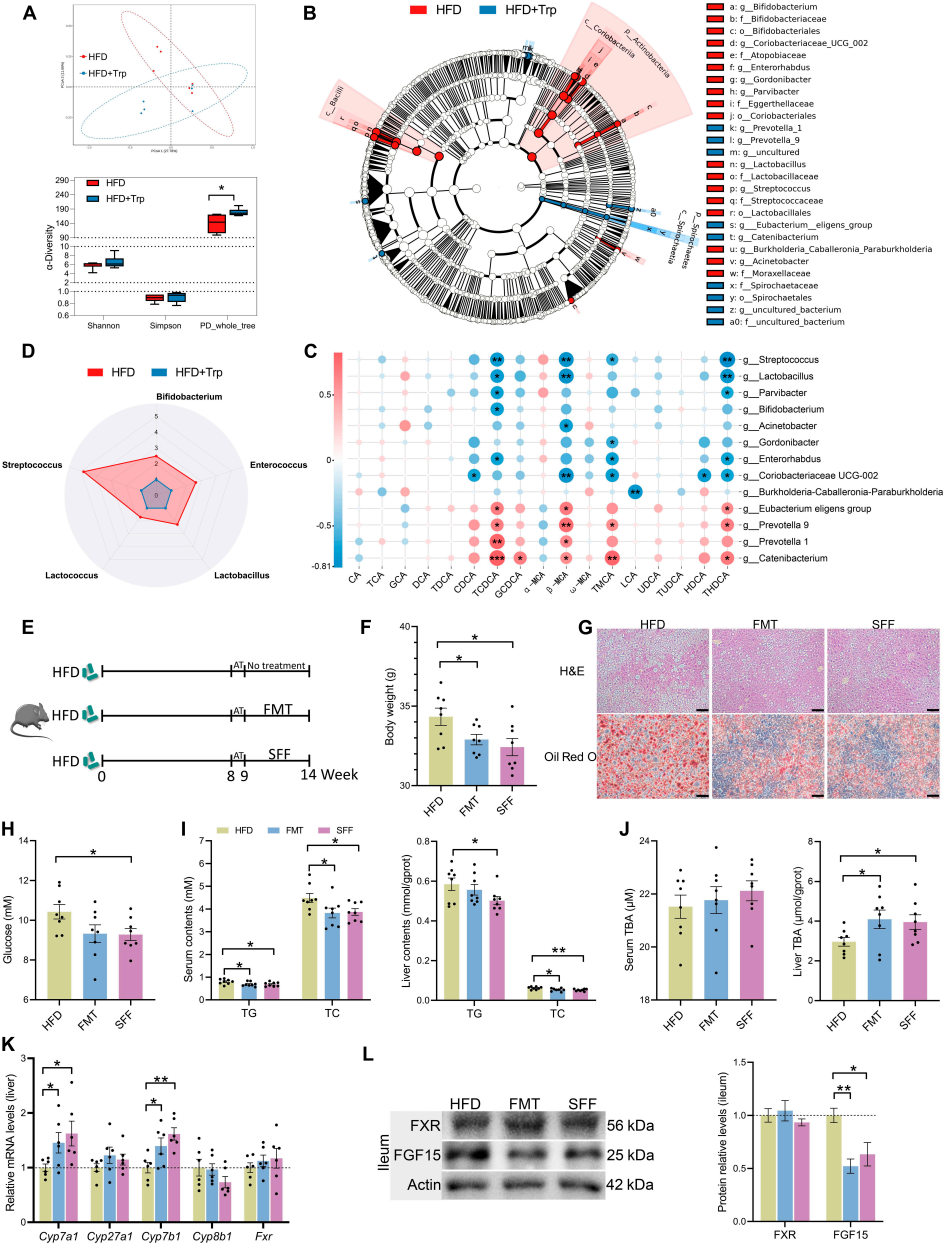
Trp ameliorates MS using the gut microbiota–BA crosstalk. (A) Scatterplot from PCoA in bacterial communities based on unweighted UniFrac distance, and alpha diversity estimates of microbiota community by Shannon, Simpson, and PD whole tree (*n* = 6 per group). (B) LEfSe analysis of intestinal bacterial communities. Circles radiating from inside to outside in the evolutionary branching diagram represent taxonomic levels from phylum to genus (or species) (*n* = 6 per group). (C) Spearman correlation between BA profile and significantly different microbes. The color of each spot in the heatmap corresponds to the *R* value of the Spearman correlation analysis between microbial abundance and BA component concentration. The correlation coefficient *r* is represented by a color. *r* > 0 means positive correlation and is represented by red; *r* < 0 means negative correlation and is represented by blue; the darker the color, the stronger the correlation (*n* = 6 per group). *P < 0.05. (D) Relative abundance of BSH-enriched microbes (*n* = 6 per group). (E) Schematic of the experimental procedure to test the role of Trp supplementation-produced gut microbiota in altering BAs and alleviating MS. (F) Body weight in the last week (*n* = 8 per group). (G) H&E and Oil Red O staining images of liver. Scale bar, 50 μm. (H) Serum glucose level (*n* = 8 per group). (I) TC and TG levels in serum and liver. (*n* = 8 per group). (J) TBA levels in serum and liver (*n* = 8 per group). (K) Liver mRNA levels of BA metabolism-related genes (*Cyp7a1*, *Cyp27a1*, *Cyp7b1*, *Cyp8b1*, and *Fxr*) (*n* = 6 per group). (L) Ileum protein expression of FXR and FGF15. Values are means ± SEM. For statistical analysis, a 2-tailed unpaired Student’s *t* test was used. *P < 0.05, **P < 0.01 versus the HFD group. AT, antibiotics.

Gut microbiota is involved in BA metabolism, interacts with BAs, and influences BA composition. Therefore, we explored the effect of Trp on the gut microbiota composition in mice with MS through 16S rDNA gene sequencing. Principal coordinates analysis (PCoA) showed a separation in the gut microbiota structure between the HFD group and the Trp group. Alpha diversity analysis, including Shannon index and Simpson index, did not show significant differences, but phylogenetic diversity (PD) whole tree was significantly higher in the Trp group than in the HFD group. This indicates that the gut microbiota structure in mice with MS was affected by Trp (Fig. 3A). The predominant phyla in the fecal microbiota were Bacteroidetes, Firmicutes, Proteobacteria, and Actinobacteria (Fig. S3). To identify specific bacterial taxa or phylotypes at the species level and to ascertain the taxonomic differences of specific bacteria between HFD-fed and Trp-supplemented mice, a linear discriminative analysis (LEfSe) was conducted. At the phylum level, the cladogram revealed that many of the different bacteria in the Actinobacteria were enriched in the HFD-fed mice. At the genus level, the cladogram demonstrated that the HFD-fed mice exhibited 9 discriminative features, while the Trp-supplemented mice exhibited 4 (Fig. 3B). Spearman correlation analysis was further performed using these genus-level differential bacteria and fecal BAs to understand gut microbiota–BA interactions. Correlation analysis revealed that many non-12-OH BAs, including TCDCA, β-MCA, TMCA, and THDCA, have a strong negative correlation with Streptococcus, Lactobacillus, Parvibacter, Bifidobacterium, Acinetobacter, Gordonibacter, Enterorhabdus, and Coriobacteriaceae UCG-002, while they have a strong positive correlation with *Eubacterium eligens* group, Prevotella 9, Prevotella 1, and Catenibacterium (*P* < 0.05) (Fig. 3C). BSH enzymes deconjugate glycine- or taurine-conjugated BAs to unconjugated BAs. Since the abundance of 3 BSH-producing bacteria, Streptococcus, Lactobacillus, and Bifidobacterium, was significantly enriched in the HFD + Trp group, we quantified the abundance of the 5 BSH-producing bacteria (Streptococcus, Lactobacillus, Bifidobacterium, Enterococcus, and Lactococcus). Radar plots showed that the HFD + Trp group formed a smaller area and that Trp reduced their abundance, which is consistent with the elevation of conjugated BAs in the liver and feces (Fig. 3D). We also examined the colonic expression of genes downstream of aryl hydrocarbon receptor (AhR) (including *Cypa1*, *Cyp1a2*, and *Cypb1*) in response to Trp. Trp treatment significantly up-regulated *Cypa1*, *Cyp1a2*, and *Cypb1* (Fig. S4), indicating AhR activation in colon tissues.

To investigate whether Trp treatment depends on the microbiota, mice were fed an HFD for 8 weeks and then gavaged with mixed antibiotic solutions for 1 week. Then, mice were orally gavaged with FMT or SFF from donor mice (donor mice were from the HFD + Trp group in the first mouse experiment), while the control group (HFD group) was orally gavaged with sterile saline. Gavage lasted for 5 weeks, during which time the HFD was maintained (Fig. 3E). As a result, the mice that received microbiota from HFD and Trp-treated mice or SFF from HFD and Trp-treated mice showed lower weight gain and serum glucose, TG, and TC concentrations compared to the HFD group. H&E and Oil Red O staining revealed smaller lipid droplets and less ballooning degeneration in the hepatocytes of FMT- or SFF-treated mice. Consistent with the pathological sections, liver TG and TC levels were also reduced after FMT and SFF (*P* < 0.05) (Fig. 3F to I). FMT and SFF alleviated MS similarly to their donors, suggesting that the ameliorative effects of Trp on MS depend on the gut microbiota. We further investigated the effects of microbiota on BA metabolism and found that liver TBA levels were greatly increased in FMT- and SFF-treated mice, although serum TBA levels remained unchanged. Consistent with the elevated liver TBA levels, the mRNA levels of BA synthesis genes *Cyp7a1* and *Cyp7b1* were considerably elevated by FMT and SFF. The mRNA level of liver *Fxr* and the protein levels of ileum FXR were unchanged, but the protein level of ileum FGF15 was markedly suppressed by FMT and SFF, indicating that FMT and SFF inhibit the intestinal FXR–FGF15 signaling pathway, enhancing de novo BA synthesis (*P* < 0.05) (Fig. 3J to L). Notably, It is worth noting that the impacts of improved MS caused by FMT and SFF depends on the microbes co-regulated by HFD and Trp, not just the Trp-regulated microbes.

The combination of these effects suggests that, under conditions of an HFD, gut microbiota play an essential and necessary role in inhibiting FXR signaling and improving BA metabolism induced by Trp. It is reasonable to assume that the gut microbiota–BA crosstalk exerts its ameliorative effects through the gut FXR–FGF15 axis.

### Trp ameliorates MS by inhibiting intestinal FXR signaling

**Fig. 4.**
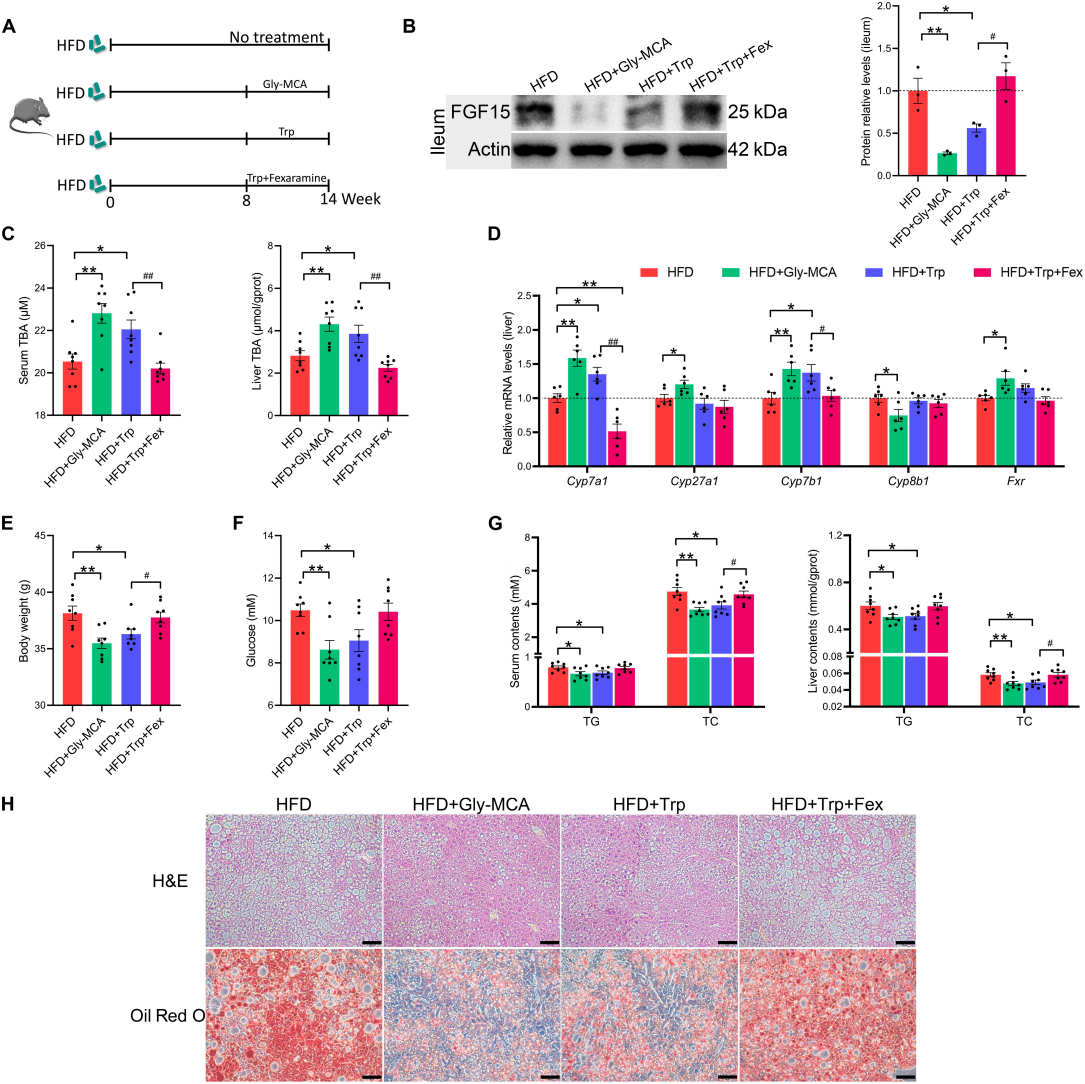
Trp ameliorates MS by inhibiting intestinal FXR signaling. (A) Schematic of the experimental procedure to test whether Trp ameliorates MS through the intestinal FXR signaling pathway. (B) Ileum protein expression of Fgf15. (C) Serum and liver TBA levels (*n* = 8 per group). (D) Liver mRNA levels of BA metabolism-related genes (*Cyp7a1*, *Cyp27a1*, *Cyp7b1*, *Cyp8b1*, and *Fxr*) (*n* = 6 per group). (E) Body weight in the last week (*n* = 8 per group). (F) Serum glucose level (*n* = 8 per group). (G) TC and TG levels in serum and liver (*n* = 8 per group). (H) H&E and Oil Red O staining images of liver. Scale bar, 50 μm. Values are means ± SEM. For statistical analysis, a 2-tailed unpaired Student’s *t* test was used. *P < 0.05, **P < 0.01 versus the HFD group. ^#^*P* < 0.05, ^##^*P* < 0.01 versus the HFD + Trp group.

To evaluate whether Trp alleviates MS through the intestinal Fxr pathway, mice were given different treatments starting from 8 weeks of HFD feeding: daily gavage of Gly-MCA (an intestine-specific FXR inhibitor), Trp intervention, and daily gavage of Fex (an intestine-specific FXR activator) in addition to the Trp intervention. The control group (HFD group) was left untreated, and the experiment lasted for 6 weeks, during which time all the mice were kept on the HFD (Fig. 4A). In the study of Fig. 1A, it can be observed that in the fourth week of the Trp intervention, there was a significant reduction in BW in the HFD + Trp group compared to the HFD group (Fig. 1B). This also means that Trp had a significant therapeutic effect on obesity after 4 weeks of intervention. Therefore, in the FXR regulation study, we changed the intervention period of Trp from 8 weeks to 6 weeks, which also ensured its therapeutic effect. In addition, the 14-week experimental period was consistent with the FMT or SFF experiment.

Western blot (WB) results confirmed that Gly-MCA treatment caused a strong inhibition of intestinal FGF15, and Trp treatment inhibited intestinal FGF15 similarly to Gly-MCA treatment, whereas Fex reversed the Trp-induced reduction in FGF15 protein levels (*P* < 0.05) (Fig. 4B). Consistent with changes in the FXR–FGF15 axis, Gly-MCA treatment and Trp treatment significantly increased serum and liver TBA levels, whereas Fex treatment reversed the Trp-induced increase in BA levels (*P* < 0.05) (Fig. 4C). In terms of gene expression levels of enzymes related to BA synthesis, Gly-MCA treatment promoted the mRNA expression levels of *Cyp7a1*, sterol 27-hydroxylas (*Cyp27a1*), and *Cyp7b1*. Trp treatment markedly promoted the mRNA expression levels of *Cyp7a1* and *Cyp7b1*, whereas Fex reversed the promotion by Trp. Notably, Gly-MCA stimulated hepatic *Fxr* gene expression, which may be due to a compensatory effect of excessive inhibition of intestinal FXR–FGF15 axis (*P* < 0.05) (Fig. 4D). In terms of amelioration of MS, both Gly-MCA and Trp reduced body weight, serum glucose, TG, and TC levels in the liver and serum. H&E and Oil Red O staining showed that both Gly-MCA and Trp inhibited hepatic lipid accumulation. However, Fex reversed the ameliorative effect of Trp on MS, suggesting that Trp alleviates MS by improving BA metabolism through inhibition of intestinal FXR signaling (*P* < 0.05) (Fig. 4E to H).

In summary, Trp inhibits intestinal FXR signaling mediated by the gut microbiota–BA crosstalk, which in turn promotes de novo BA synthesis, thereby ameliorating MS.

### Trp inhibits intestinal FXR signaling and improves lipid metabolism in finishing pigs

**Fig. 5.**
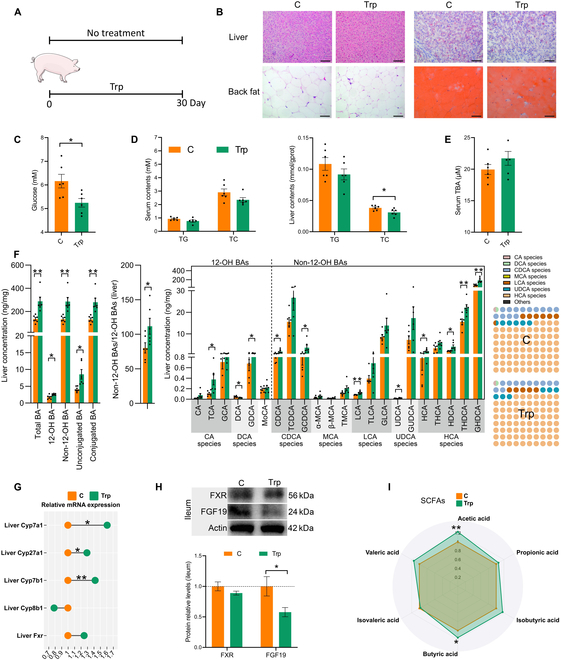
Trp supplementation promotes BA synthesis in finishing pigs. (A) Schematic of the experimental procedure to test the role of Trp in finishing pigs. (B) H&E analyses of liver and back fat. Scale bar, 100 μm. Oil Red O staining of liver and back fat. Scale bar, 100 μm. (C) Serum glucose level. (D) Serum and liver TC and TG levels. (E) Serum TBA level. (F) Liver BA profile of finishing pigs. (G) Liver mRNA levels of BA metabolism-related genes (*Cyp7a1*, *Cyp27a1*, *Cyp7b1*, *Cyp8b1*, and *Fxr*). (H) Ileum protein expression of FXR and Fgf19. (I) SCFA level. Values are means ± SEM (*n* = 6 per group). For statistical analysis, a 2-tailed unpaired Student’s *t* test was used. *P < 0.05, **P < 0.01 versus the control (C) group.

Pigs are less susceptible to metabolic diseases due to their high levels of the unique beneficial BAs, HCA species. We next investigated the effect of Trp on BA metabolism in a pig model. Therefore, we chose the finishing pig model to further validate the role of Trp in improving lipid metabolism. We included 0.78% Trp in the basal diet of finishing pigs for 30 days and observed changes in growth performance as well as lipid metabolism (Fig. 5A). There were no significant differences in the initial and final weights between the 2 groups of finishing pigs. There were also no noticeable distinctions in average daily feed intake, average daily gain, and feed conversion ratio. However, Trp significantly reduced back fat thickness (*P* < 0.05) (Table). H&E and Oil Red O staining showed that Trp inhibited lipid accumulation in back fat, whereas this effect was not evident in the liver (Fig. 5B). Trp also reduced serum glucose and liver TC concentrations in finishing pigs (*P* < 0.05) (Fig. 5C and D).

**Table.** Growth performance of finishing pigs

	Control	Trp	*P* value
Initial body weight (kg)	102.02 ± 2.08	104.35 ± 3.23	0.56
Final body weight (kg)	132.73 ± 3.10	131.73 ± 1.49	0.78
Average daily feed intake (kg/day)	2.87 ± 0.26	2.71 ± 0.18	0.64
Average daily gain (kg/day)	1.02 ± 0.05	0.91 ± 0.06	0.19
Feed conversion ratio	2.84 ± 0.32	3.07 ± 0.37	0.65
Back fat thickness (cm)	3.32 ± 0.21	2.32 ± 0.21	**<0.01**

The data are presented as the mean ± SEM. Boldface indicates a significant *P* value.

Next, BA metabolism was measured. Trp had no significant effect on serum TBA but caused a significant increase in hepatic TBA. Trp markedly elevated the levels of numerous BA components in liver tissue, including taurocholic acid (TCA), DCA, glycodeoxycholic acid (GDCA), CDCA, glycochenodeoxycholic acid (GCDCA), lithocholic acid (LCA), ursodeoxycholic acid (UDCA), HCA, HDCA, THDCA, and GHDCA. Overall, 12-OH BAs, non-12-OH BAs, unconjugated BAs, conjugated BAs, and the ratio of non-12-OH to 12-OH BAs were significantly higher in Trp-supplemented finishing pigs (*P* < 0.05). Moreover, the proportion of HCA species has increased (Fig. 5E and F). Consistent with increased hepatic BA levels was a significant up-regulation of BA synthesis genes in the liver, including *Cyp7a1*, *Cyp27a1*, and *Cyp7b1*. The protein expression levels of intestinal FXR were unchanged, whereas FGF19 was significantly suppressed by Trp (*P* < 0.05) (Fig. 5G and H). The protein expression of liver FGF19 was also decreased (*P* < 0.05) (Fig. S2B), suggesting that the enhancement of hepatic BA synthesis is mediated by the intestinal FXR–FGF19 axis. Furthermore, Trp increased fecal TBA levels (Fig. S5), which is consistent with increased fecal TBA levels in the mouse model. This shows that Trp increases both hepatic BA synthesis and BA excretion in mice and finishing pigs. The levels of short-chain fatty acids (SCFAs) in the colonic contents were also measured. Radar plots showed that Trp remarkably raised levels of acetic acid and butyric acid, demonstrating that Trp improved the intestinal environment (*P* < 0.05) (Fig. 5I).

**Fig. 6.**
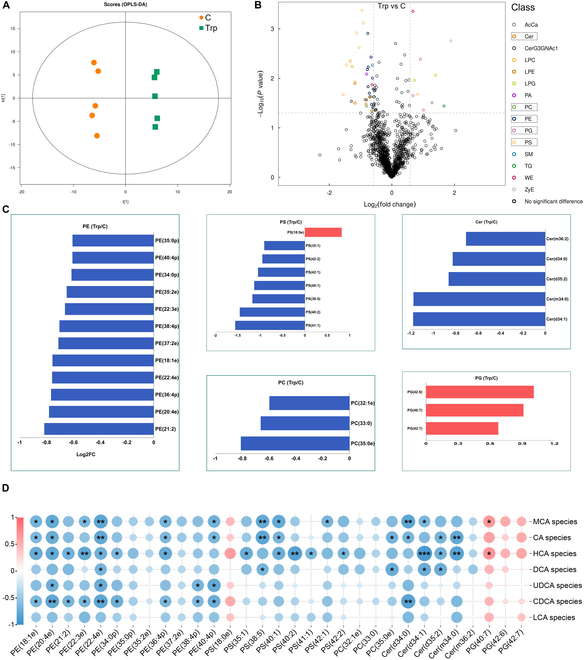
Lipidomics reveals that Trp alters the lipid profile of the liver in finishing pigs. (A) OPLS-DA of each group (*n* = 5 per group). (B) Changes of lipid molecules in the liver. FC > 1.5, *P* < 0.05 are significantly up-regulated lipid molecules. FC < 0.67, *P* < 0.05 are significantly down-regulated lipid molecules. Different colors represent the classification of lipid molecules. (C) Lipid molecules with significant differences. Blue color represents down-regulation, and red color represents up-regulation. (D) Spearman correlation between BA species and significantly different lipid molecules. The color of each spot in the heatmap corresponds to the *R* value of the Spearman correlation analysis between microbial abundance and BA component concentration. The correlation coefficient *r* is represented by a color. *r* > 0 means positive correlation and is represented by red; *r* < 0 means negative correlation and is represented by blue; the darker the color, the stronger the correlation (*n* = 5 per group). *P < 0.05.

To investigate the influence of Trp on the hepatic lipid profile, we employed lipidomics to analyze the lipid profile of the liver. The total lipid concentration did not differ significantly (Fig. S6A). Orthogonal partial least squares discriminant analysis (OPLS-DA) revealed distinct lipid profiles between the 2 groups of pigs (Fig. 6A). A comparison of the levels of various lipid classes showed significant increases in wax esters (WEs) and zymosterol (ZyE) due to Trp supplementation (*P* < 0.05) (Fig. S6B and C). The volcano plot depicts changes in lipid molecules in the liver. Ten lipid molecules were significantly up-regulated [fold change (FC) > 1.5, *P* < 0.05], while 34 lipid molecules were significantly down-regulated (FC < 0.67, *P* < 0.05) by Trp. The different colors represent the classification of the lipid molecules, where the number of significantly different lipid molecules is higher in phosphatidylethanolamine (PE), phosphatidylserine (PS), phosphatidylcholine (PC), phosphatidylglycerol (PG), and ceramides (Cer) (Fig. 6B). PE, PS, PC, and PG are all glycerophospholipids (GPs). Twelve lipid molecules in PE, 7 in PS, 3 in PC, and 5 in Cer were clearly down-regulated. Additionally, 1 lipid molecule in PE and 3 in PG were clearly up-regulated (Fig. 6C). In order to characterize the intra-correlation of the same lipid and the correlations among the lipids of different classes, we analyzed the correlations of altered lipids in the liver. The results show that PE and PC have strong internal correlations not only within themselves but also with other different types of lipid molecules (Fig. S6D). Spearman correlation analysis was further conducted using these lipid molecules and hepatic BA species to explore the relationship between BAs and lipids in the liver. Correlation analysis showed that MCA species, CA species, HCA species, and DCA species are strongly negatively correlated with several PE, PS, PC, and Cer molecules but positively correlated with PG molecules (*P* < 0.05) (Fig. 6D). This suggests that BAs from these species are closely related to the hepatic lipid profile, particularly HCA species.

In conclusion, these data demonstrate that Trp inhibits intestinal FXR signaling and enhances BA synthesis, which in turn has a beneficial effect on lipid metabolism in finishing pigs.

## Discussion

Trp has a major influence on the host microbes and a broad array of metabolic processes and has the potential to improve MS. MS, characterized by disrupted energy balance and dysregulated lipid metabolism, can lead to various complications such as obesity and hepatic steatosis [24]. Interactions between host lipid metabolism and gut microbes are mediated through microbial metabolites. Among the numerous microbial metabolites, BAs constitute a highly abundant pool of host-derived and microbially modified metabolites that play crucial roles in regulating host lipid homeostasis [25,26].

**Fig. 7.**
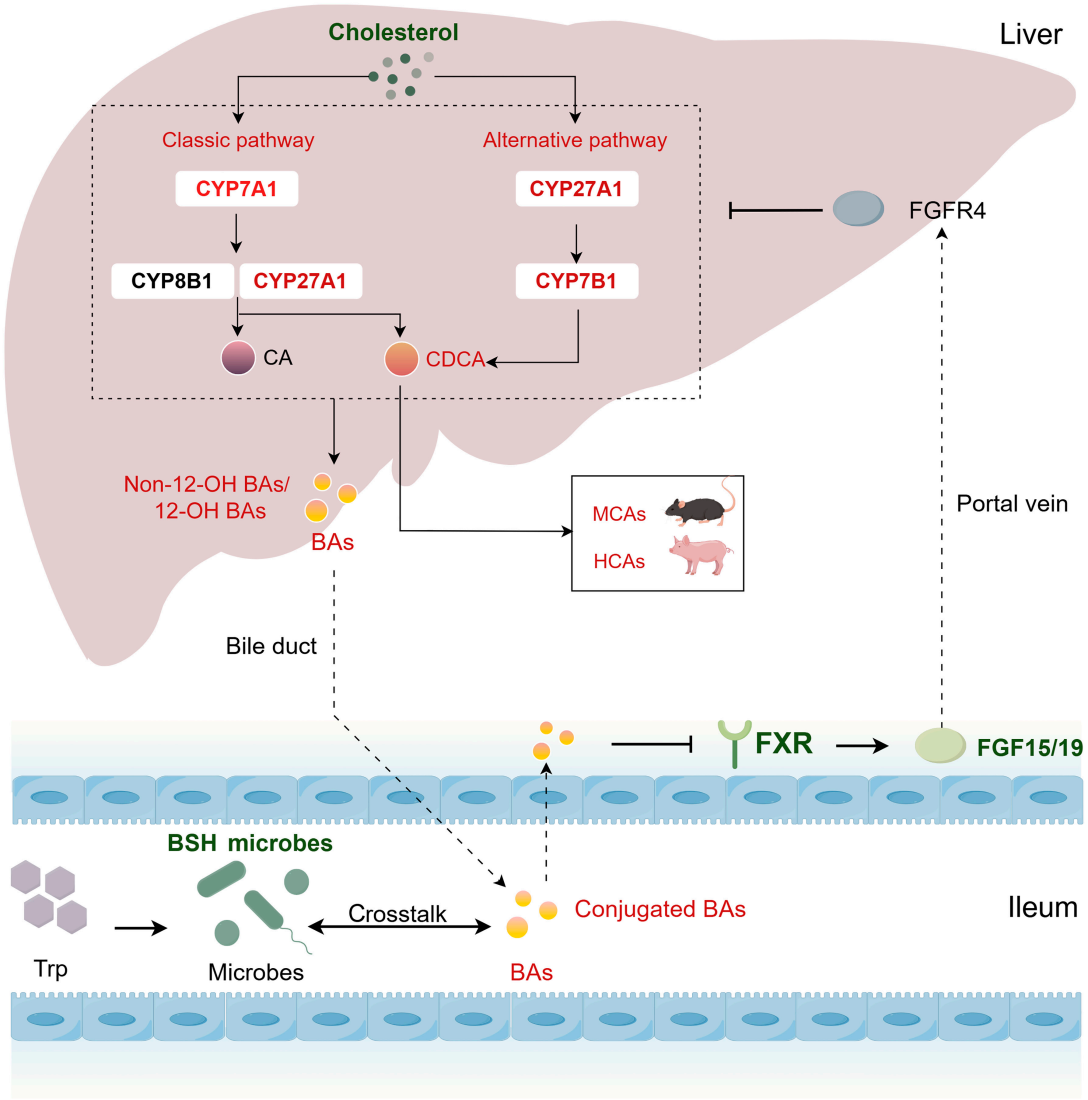
The mechanism of Trp in alleviating MS. Trp altered the composition of the gut microbiota and reduced the abundance of BSH-producing bacteria in mice, leading to a decrease in bacterial deconjugation capacity and consequently an increase in the total amount of conjugated BAs. The accumulation of conjugated BAs directly inhibits FXR–FGF15 signaling. Reduced FGF15/19 acts on FGFR4 via the hepatic portal vein, which led to up-regulation of hepatic BA synthase CYP7A1, CYP7B1, and CYP27A1 expression and increased hepatic synthesis of BAs from scratch. The classical pathway of BA synthesis is initiated by CYP7A1 and results in the production of CA and CDCA through the subsequent action of CYP8B1 and CYP27A1. The alternative pathway of BA synthesis is initiated by CYP27A1 and produces CDCA via CYP7B1. In the rodent liver, the majority of CDCA is converted to MCA. In the pig liver, CDCA is primarily converted to HCA. Elevated ratio of non-12-OH to 12-OH BAs in both pigs and mice, increased MCA species in mice, and increased HCA species in pigs indicated the importance of the BA alternative pathway. Hepatic TC consumption increased and lipid metabolism improved as a result of increased BA levels. The altered BA levels and composition, in turn, affect the composition of the microbiota, with crosstalk between gut microbiota and BA regulating metabolism. The red color in the picture represents up-regulation. The green color in the picture represents downregulation or inhibition.

BAs are amphipathic steroid molecules that are synthesized from cholesterol, with 2 primary biosynthetic pathways. The classical pathway produces mainly 12-OH BAs and its conjugates, whereas the alternative pathway produces mainly non-12-OH BAs and its conjugates [27]. The classical pathway is initiated by CYP7A1 and results in the production of CA and CDCA through the subsequent action of CYP8B1 and CYP27A1. The alternative pathway is initiated by CYP27A1 and produces CDCA via CYP7B1. In the rodent liver, the majority of CDCA is converted to MCA. In the pig liver, CDCA is primarily converted to HCA [28]. In this study, the levels of CDCA and its transformed MCAs and HCAs are elevated, indicating the promotion of alternative pathways (Fig. 7). Up-regulation of the alternative pathway of BA synthesis has the effect of increasing the production of hydrophilic BAs such as UDCA and MCA, leading to reduced intestinal cholesterol and fat absorption [29]. Up-regulation of the alternative pathway also increases the ratio of non-12-OH to 12-OH BAs, which beneficially reduces lipid uptake [30]. Recent studies have demonstrated that up-regulation of the alternative pathway finely regulates cholesterol, lipid, carbohydrate, and energy homeostasis [31]. Increased cholesterol catabolism via the alternative pathway resulted in rapid and marked reduction in hepatic TGs [32]. In mice with type 2 diabetes, decreased CYP7B1 mRNA expression has been observed [33,34]. Therefore, enhancement of the alternative pathways promotes improvement in lipid metabolism. Our results demonstrate that Trp enhances BA synthesis in both the classical and alternative pathways in both mice and pig models. Moreover, liver BA profiles exhibited increased ratio of non-12-OH to 12-OH BAs. There is increasing evidence that hepatic TGs are not the determining factor of lipotoxicity. Certain lipid classes, such as cholesterol and Cer, act as disruptors in the liver [35]. Thus, enhancement of BA synthesis depletes cholesterol in the liver, reducing liver toxicity, and decreasing certain ceramide molecules in pig liver alleviates the burden on the liver.

In the current study, the gut microbiota is reconstituted in Trp-treated HFD mice, while both liver and fecal BA profiles are modified. Spearman correlation analysis indirectly implies a positive or negative relationship between fecal BAs and differential genera by Trp treatment. Trp also reduces the abundance of BSH-producing bacteria. This resulted in decreased bacterial deconjugation capacity and an increase in the total amount of conjugated BAs. The accumulation of taurine-conjugated BAs directly inhibits the intestinal FXR–FGF15 signaling pathway [13]. FMT and SFF indicate a direct impact of gut microbes in influencing BA signaling. The observation that the gut-specific inhibitor Gly-MCA mimicked Trp’s effects, and that these effects were reversed by the gut-specific activator Fex, provides additional evidence that Trp interacts with intestinal FXR. These results suggest that gut microbes influence BA profiles and inhibit the intestinal FXR–FGF15/19 axis, which plays a crucial role in Trp’s amelioration of MS. However, altered BA metabolism also affects the gut microbes. The interactions between the gut microbiota and BAs are reflected not only in the influence of the microbiota on BA metabolism but also in the role of BAs in shaping the structure and function of the gut microbiota. BAs can promote the growth of bacteria that metabolize BAs and inhibit the growth of BA-sensitive bacteria, thus maintaining bacterial homeostasis, inhibiting bacterial overgrowth in the small intestine, protecting the intestinal barrier function, and inhibiting bacterial translocation [36,37]. In the present study, the inhibition of intestinal FXR signaling by Trp resulted in increased hepatic BA synthesis. The mRNA expression of the BA transport genes, *Bsep* and *Abst*, was promoted. These represent Trp-driven enhancement of BA metabolism. Secretion of BAs can provide enough energy to support a large diversity of microorganisms, with elevated alpha diversity in the Trp-treated mice in this study. In addition to compositional changes, BAs can improve the functional capacity of the microbiota [38].

Notably, the levels of TMCA and HDCA increased in the feces and liver of mice. TMCA has been identified as an FXR inhibitor [12], while HDCA has been shown to improve MASLD due to its inhibitory effect on FXR [39]. Pigs exhibit a unique high proportion of HCA species. These HCA species have numerous benefits, including improved glucose metabolism and reduced diabetes risk [40]. Trp supplementation increases HCA species content in finishing pigs. Ultimately, Trp improves lipid metabolism and alters numerous GP molecules in the liver. Specifically, Trp reduces PE and PC levels, which are the most abundant phospholipids in all mammalian cell membranes [41]. Trp also increased the levels of SCFAs in the pig intestine. SCFAs are nondirect nutrients produced by intestinal microorganisms and have significant physiological regulatory functions, such as providing a portion of the body’s energy requirements and regulating electrolyte balance, protecting the intestinal mucosal barrier, facilitating the absorption of nutrients, regulating lipid metabolism, modulating pH in the intestines, inhibiting intestinal inflammation, and modulating immune responses [42]. Therefore, Trp supplementation improved the overall metabolism of the finishing pigs.

Furthermore, inhibition of intestinal FXR signaling offers numerous benefits beyond enhancing BA synthesis. Inhibition of the intestinal FXR–SHP/FGF19 signaling pathway led to decreased expression of hepatic lipid synthesis-related genes such as sterol regulatory element-binding protein 1c (SREBP-1c), fatty acid synthase, stearoyl coenzyme A (CoA) desaturase-1, and acetyl CoA carboxylase [43]. This reduction in hepatic lipid synthesis improved overall glucose tolerance and ameliorated fatty liver. Our results also demonstrated that Trp reduced the levels of certain Cer molecules in pig liver. Elevated levels of Cer have been linked to the development of metabolic diseases. Cer has been shown to induce lipotoxicity in the liver by increasing endoplasmic reticulum stress and fatty acid synthesis through the SREBP-1 signaling pathway. Additionally, Cer has been demonstrated to impair adipose function, leading to a reduction in the ratio of beige to white adipocytes [44], whereas tauro-β-muricholic acid (TβMCA) and Gly-MCA, inhibitors of intestinal FXR, reduce levels of Cer in the gut and tissues. Reduced Cer inhibits de novo hepatic lipid synthesis via SREBP-1c [14] and promotes browning of WAT as well as thermogenesis of brown adipose tissue [45].

In addition, Trp is converted by the gut microbiota into a variety of metabolites that are also useful in improving MS. The Trp catabolites include indole, tryptamine, indoleethanol (IE), indolepropionic acid (IPA), indolelactic acid (ILA), indoleacetic acid (IAA), skatole, indolealdehyde (IAld), and indoleacrylic acid (IA) and can affect host physiology in a variety of ways. Some indoles and related molecules bind to the AhR on the gut surface. The AhR plays a key role in homeostasis not only in the gut but also elsewhere in the gut microbiota and in the host body. AhR signaling plays many important roles in the gut and overall health, including maintaining gut barrier function, regulating the composition of gut microbiota, maintaining gut immune cell populations and reducing inflammation, activating detoxification pathways, and supporting nervous system health [46]. Reduced intestinal AhR activity has been observed in a variety of chronic diseases, including obesity, MS, hypertension, and atherosclerosis [19]. In the present study, Trp supplementation resulted in colonic AhR activation, suggesting that Trp metabolism influences the gut microbiota and activates the AhR to produce beneficial effects. The effect of Trp on inhibiting intestinal FXR signaling, regulating BA metabolism, and improving MS in the present study may be due to the action of specific metabolites produced by Trp, which should be further investigated in future studies.

Therefore, Trp alters the intestinal microbiota and reduces the relative abundance of BSH-enriched microbes, leading to an elevation of conjugated BAs and consequent inhibition of the FXR–FGF15/19 axis. Reduced FGF15/19 acts on FGFR4 via the hepatic portal vein, which led to up-regulation of hepatic BA synthases CYP7A1 (classical pathway), CYP7B1, and CYP27A1 (alternative pathway), thereby increasing de novo hepatic BA synthesis. Elevated ratio of non-12-OH to 12-OH BAs in both pigs and mice, increased MCA species in mice, and increased HCA species in pigs indicated the importance of the BA alternative pathway. Increased hepatic TC consumption and improved lipid metabolism resulted from elevated BA levels. The altered BA levels and composition, in turn, affect the composition of the microbiota, with crosstalk between gut microbiota and BA regulating metabolism (Fig. 7).

Taken together, our findings support that Trp ameliorates MS by inhibiting intestinal FXR signaling mediated through the gut microbiota–BA crosstalk. These findings provide compelling evidence for the efficacy of Trp in improving lipid metabolism.
