# Supplementary material for: Tryptophan Ameliorates Metabolic Syndrome by Inhibiting Intestinal Farnesoid X Receptor Signaling: The Role of Gut Microbiota–Bile Acid Crosstalk
Source: Research (Wash D C). 2024 Dec 13;7:0515. doi: 10.34133/research.0515 (PMC11638488; doi:10.34133/research.0515)
Supplement: Supplementary 1 — Materials and Methods Figs. S1 to S6 Tables S1 and S2 [file research.0515.f1.docx]

**Supplementary Information**

**Tryptophan Ameliorates Metabolic Syndrome by Inhibiting Intestinal FXR Signalling: The Role of Gut Microbiota-Bile Acid Crosstalk**

Jiayi Chen**^#^**, Hao Yang**^#^**, Yingjie Qin, Xinbo Zhou, Qingquan Ma*

College of Animal Science and Technology, Northeast Agricultural University, Harbin, 150030, China

^#^These authors have contributed equally

*To whom correspondence should be addressed. E-mail: maqingquan@neau.edu.cn (Q. Ma)

Tel: +86 451 55191395.

**Materials and methods**

**Glucose and insulin tolerance tests**

A glucose tolerance test (GTT) was conducted after a 12-hour fast at the end of week 14, and an insulin tolerance test (ITT) was conducted after a 6-hour fast at the end of week 15. GTT and ITT were conducted by intraperitoneal injection of glucose (2 g/kg) and insulin (0.75 U/kg) solution to study systemic glucose homeostasis. Tail vein blood glucose concentrations were measured using a glucose meter (On Call, Hangzhou, China) at 0, 15, 30, 60, 90, and 120 minutes after injection of glucose or insulin. The GTT and ITT curves and areas under the curves (AUC) were analyzed using GraphPad Prism 9.

**Samples collection**

At the end of the intervention, the mice were subjected to overnight food deprivation and subsequently euthanized using gentle ether anesthesia. Blood samples were collected from the orbital vein of the mice, centrifuged at 3000×g for 15 minutes at 4°C, and stored at -80°C until analysis. Various organs were rapidly removed and weighed. Part of the liver and white adipose tissue (WAT) were fixed in 4% paraformaldehyde for histological examination, and the remaining tissues were immediately frozen in liquid nitrogen and stored at -80°C until analysis.

The pigs were slaughtered after supplementation, and final body weight (BW), average daily gain (ADG), and average daily feed intake (ADFI) data were collected. Blood samples were obtained from the jugular vein and centrifuged at 3000×g for 15 minutes at 4°C to collect the serum. The serum was stored at -80°C for further study. The liver, ileum, and back fat tissues were rapidly removed. Portions of the tissues were immediately fixed in 4% formaldehyde for histological examination, and the remaining portions were stored at -80°C until further use.

**Chemical analysis**

Glucose (GLU), triglycerides (TG), total cholesterol (TC), aspartate transaminase (AST), alanine transaminase (ALT) and total bile acids (TBA) were measured using commercially available diagnostic kits (Nanjing Jiancheng Bioengineering Institute, Nanjing, China).

**Histological examination**

After fixation with 4% formaldehyde, liver, WAT, ileum, and back fat tissues were dehydrated and embedded in paraffin. Consecutive serial tissue sections (5 µm) were cut. After the sections were deparaffinized with xylene and washed with serial dilutions of ethanol, they were assessed by routine hematoxylin-eosin (H&E) staining. Frozen sections were stained with Oil Red O. Images were observed and photographed using a light microscope (BX46 and BX53, OLYMPUS, Japan).

**Targeted bile acid metabolomics**

A 100 mg liver or feces sample (n = 6) was taken, and 500 μL of pre-cooled 90% methanol was added. The mixture was vortexed and incubated at -20°C for 20 minutes to precipitate proteins. The samples were centrifuged at 14,000 g for 15 minutes at 4°C, and 400 μL of the supernatant was dried under vacuum. 100 μL of methanol-water (1:1, v/v) was added for reconstitution, and the supernatant was separated on a Waters ACQUITY UPLC I-Class system. Chromatographic separation was performed using an Acquity BEH C18 column (1.7 µm, 2.1 mm × 100 mm, Waters). The mobile phase consisted of 0.1% formic acid (Honeywell, 94318) in aqueous solution and methanol (Fisher Chemical, A452-4). The column temperature was 45°C, the flow rate was 300 µL/min, and the sample uptake was 2 µL. Electrospray ionization (ESI) in negative ion mode was used for detection, and a 5500 QTRAP mass spectrometer (AB SCIEX) was used for mass spectrometric analysis.

**Quantitative real-time PCR analysis**

Total RNA was extracted using Trizol reagent (Thermo Fisher Scientific, MA, USA) and reverse-transcribed into cDNA using a cDNA kit (Takara, Dalian, China). Quantitative PCR assays were performed using the cDNA templates, specific primers, and SYBR Green qPCR Master Mix (Takara, Dalian, China) on an ABI PRISM 7500 SDS thermal cycler (Applied Biosystems, CA, USA). Calculations were performed using the comparative 2^−ΔΔCt^ method, and β-actin was used as an internal control. Primer sequences for each gene are listed in **Supplementary Table 1**.

**Western blot analysis**

Liver tissues were homogenized in RIPA buffer (Beyotime Biotechnology, Shanghai, China) with PMSF (Beyotime Biotechnology, Shanghai, China), and the supernatant was collected after centrifugation. Protein concentrations were determined using a BCA kit (Beyotime Biotechnology, Shanghai, China). Equivalent protein samples were separated on 12% SDS-PAGE gels and then transferred to polyvinylidene fluoride (PVDF) membranes (Bio-Rad, California, USA) by electrophoretic transfer. The membranes were blocked with 5% BSA (Biotopped, Beijing, China) for 2 hours at room temperature. The membranes were then incubated with primary antibodies overnight at 4°C. After thorough washing three times for 5 minutes each, the membranes were incubated with appropriate horseradish peroxidase (HRP)-conjugated secondary antibodies for 1 hour at room temperature. Signals were detected using a chemiluminescence imaging system. Stripping buffer was used to strip the immunoblot for reprobing. The intensity of the bands was normalized to β-actin. The primary antibodies used are listed in **Supplementary Table 2**.

**Short-chain fatty acids determination**

The SCFAs content (acetic acid, propionic acid, isobutyric acid, butyric acid, isovalerate acid and valerate acid) in the colonic contents were determined by high performance gas chromatography. The chromatographic column was an HP-INNOWAX 19091N-1331 (30.0 m/0.250 mm/0.25 µm) (Agilent, USA). The SPL temperature was 220°C, pressure was 90 kPa, total flow was 71 mL/min, purge flow was 3 mL/min; column box temperature was 180°C; FID temperature was 240°C, tail blow was 30 mL/min, hydrogen gas was 40 mL/min, and air was 400 mL/min.

**Lipidomics analysis**

Liver samples (25 mg) from each group were collected and placed in tubes. The tissues were homogenized in a 500 μL mixture of MTBE/MeOH (5:1), vortexed, and subsequently centrifuged at 3000 rpm for 15 min at 4 °C. After centrifugation, 300 μL of supernatant from each group was evaporated to dryness in a vacuum concentrator at 37 °C. The resulting lipid extracts (200 μL) were redissolved in a mixture of 50% methanol in dichloromethane, followed by sonication on ice for 10 min. Subsequently, 75 μL of the supernatant was used for analysis. Hepatic lipidomics analysis was conducted using an ultrahigh-performance liquid chromatography (UHPLC) system (Shim-pack UFLC SHIMADZU CBM30A). Samples (2 μL) were injected onto a Kinetex C18 column (2.1 × 100 mm, 1.7 μm; Thermo Fisher, MA, USA). The mobile phases consisted of water/acetonitrile (40:60, v/v) (solvent A) and acetonitrile/isopropanol (10:90, v/v) (solvent B), both containing 10 mM ammonium formate. The elution gradient was programmed as follows: starting from 40% B, increased to 100% B over 12 min, maintained at 100% B for 1.5 min, then returned to 40% B from 13.5 to 18 min. Qualitative and quantitative analyses were conducted by Applied Protein Technology Co., Ltd (Shanghai, China).

**Supplementary Tables**

**Supplementary Table 1. Mice RT-PCR primer sequences**

| Gene | Forward Sequence (5’-3’) | Reverse Sequence (5’-3’) |
| --- | --- | --- |
| *β-actin* | CAGGCATTGCTGACAGGATG | TGCTGATCCACATCTGCTGG |
| *Cyp7a1* | AGCAGCCTCTGAAGAAGTGAATGG | AGAGCCGCAGAGCCTCCTTG |
| *Cyp27a1* | CACCGATGGCTGAGGAAGAAAGAG | ACCCAGGCAAGACCGAACCC |
| *Cyp7b1* | AGCCCTGCGTGACGAAATTGAC | GAGCACAGCCTCAGAACCTCAAG |
| *Cyp8b1* | GATGGCACCCGGAAAGTGGA | TAGTGGTGGATCTTCTTGCC |
| *Fxr* | ACAGAGAGGCGGTGGAGAAGC | TCAGCGTGGTGATGGTTGAATGTC |
| *Shp* | TCTCTTCCTGCTGGGGTTGGC | ACCGCTGCTGGCTTCCTCTAG |
| *Fgf15* | GAGGACCAAAACGAACGAAATT | ACGTCCTTGATGGCAATCG |
| *Bsep* | GTTCACGGAGCTTGAGTTG | AAAAGCAGCCACTGTTCG |
| *Ntcp* | CCCTAATGGCCTGAACTCT | CTGCTGTTGAAGGTTTGCT |
| *Asbt* | CTGGGTTTCTTCCTGGCTA | GTTTCCAAGGCTACTGTTCG |
| *Cyp1a1* | GGAAGTGGAAGGGCATAGGCA | TCCAAGGCAGAATACGGTGAC |
| *Cyp1a2* | GCTTCTCCATAGCCTCGGAC | CTGGCTGACTGGTTCGAAGT |
| *Cyp1b1* | CTGGACAAGGACGGCTTCAT | ACAGTTCCTCACCGATGCAC |

**Supplementary Table 2. Antibodies for Western blot analysis**

| **Antibody** | **Cat No.** | **Manufacturer** |
| --- | --- | --- |
| β-ACTIN | AF5003 | Beyotime |
| CYP7A1 | DF2612 | Affinity Biosciences |
| CYP27A1 | DF3571 | Affinity Biosciences |
| CYP7B1 | 24889-1-AP | Proteintech |
| FXR | 25055-1-AP | Proteintech |
| SHP | DF6648 | Affinity Biosciences |
| FGF15 | sc-514647 | Santa Cruz Biotechnology |
| FGF19 | DF2651 | Affinity Biosciences |

**Supplementary Figures**

**Figure S1**

**
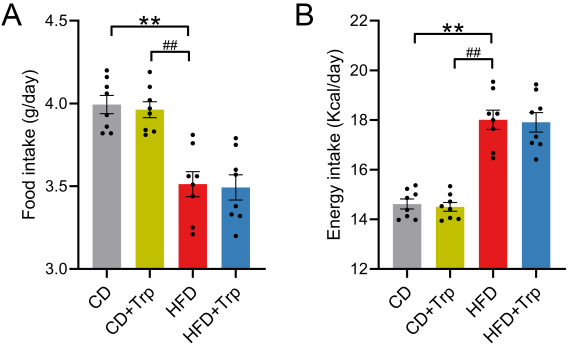
**

**Figure S1.** (**A**) Food intake. (**B**) Energy intake. Values are means ± SEM (n = 8 per group). For statistical analysis, a two-tailed unpaired Student's t-test was used. *P < 0.05, **P < 0.01 vs. the CD group. #P < 0.05, ##P < 0.01 vs. the HFD group. Chow diet (CD); high-fat diet (HFD)

**Figure S2**


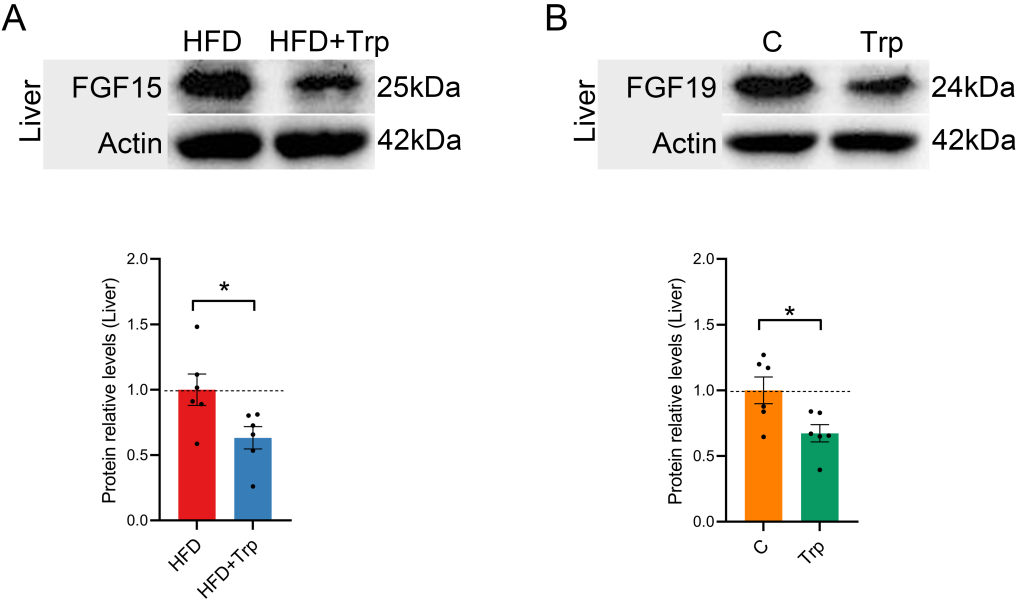


**Figure S2.** (**A**) Protein expression of liver FGF15 in mice. (**B**) Protein expression of liver FGF19 in finishing pigs. Values are means ± SEM (n = 6 per group). For statistical analysis, a two-tailed unpaired Student's t-test was used. *P < 0.05, **P < 0.01 vs. the HFD or C group. High-fat diet (HFD); fbroblast growth factor 15/19 (Fgf15/19)

**Figure S3**


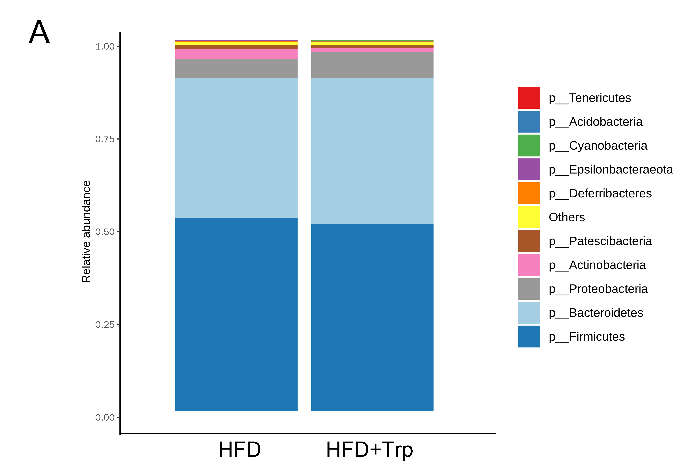


**Figure S3.** (**A**) Histogram of the relative abundance of species at the level of the phylum.

**Figure S4**

**
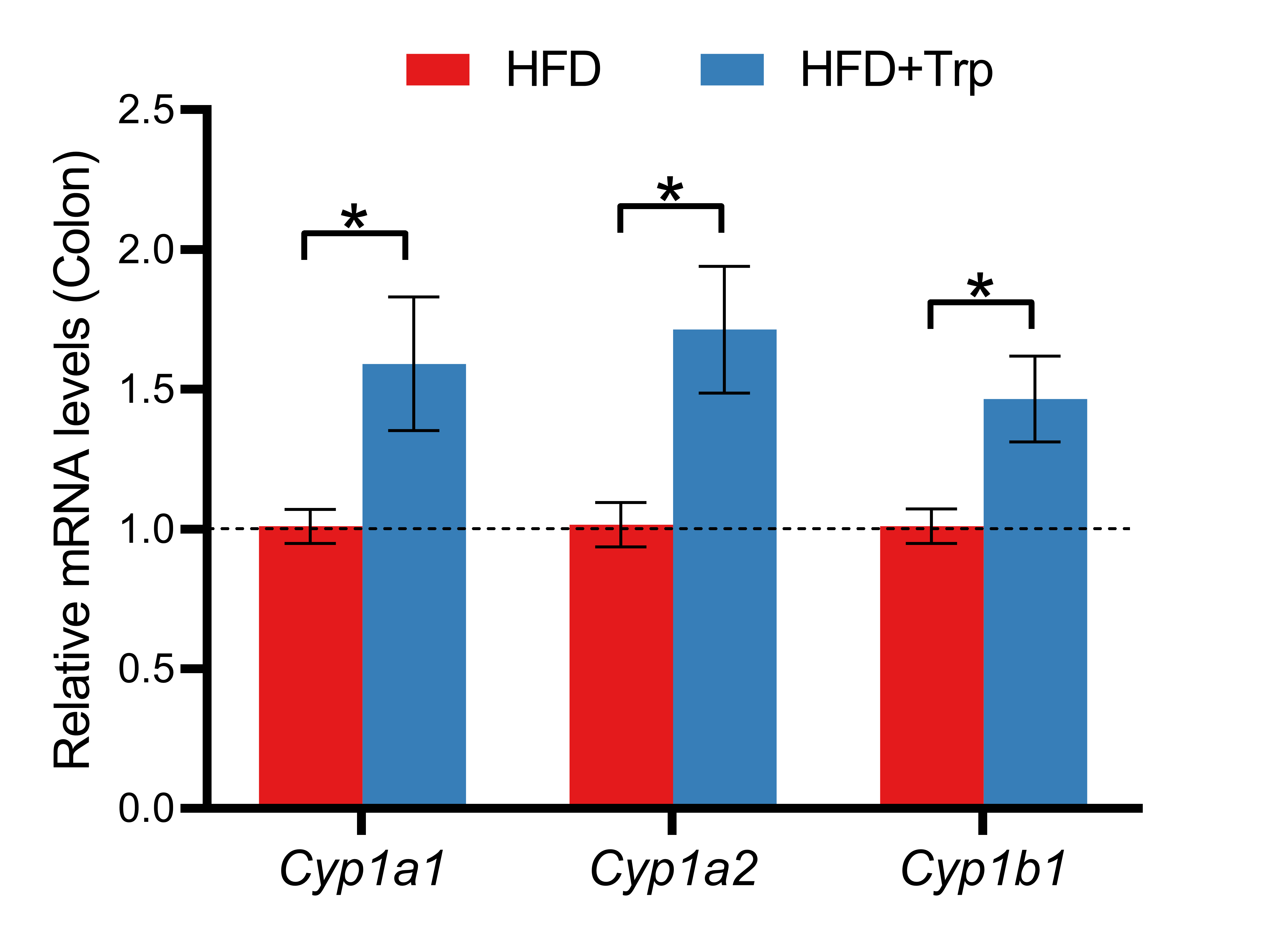
**

**Figure S4.** Colon mRNA levels of BA metabolism-related genes (*Cyp1a1*, *Cyp1a2*, *Cyp1b1*) (n = 6 per group). *P < 0.05, **P < 0.01 vs. the HFD group. High-fat diet (HFD)

**Figure S5**


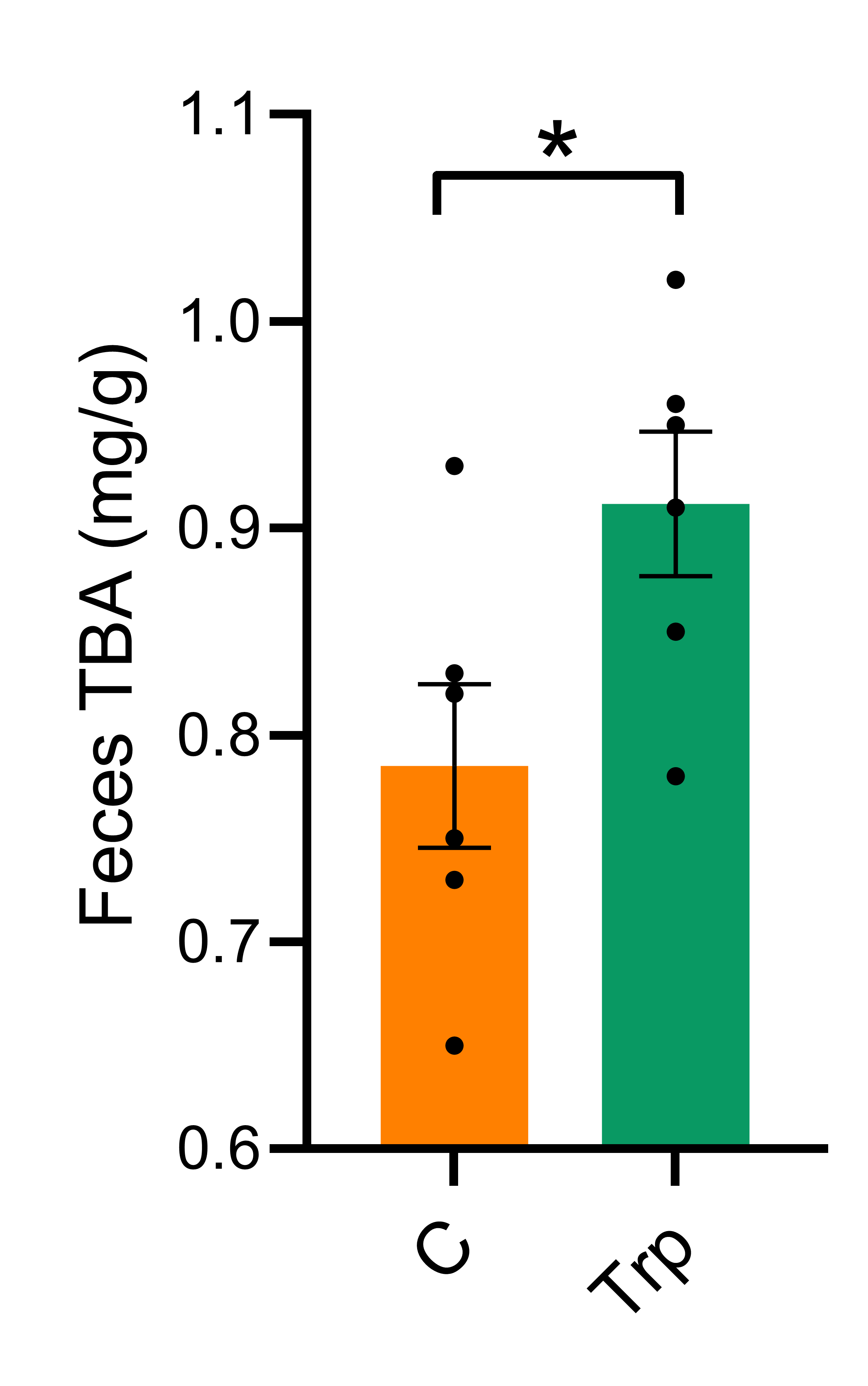


**Figure S5.** The feces TBA level. Values are means ± SEM (n = 6 per group). For statistical analysis, a two-tailed unpaired Student's t-test was used. *P < 0.05, **P < 0.01 vs. the C group

**Figure S6**


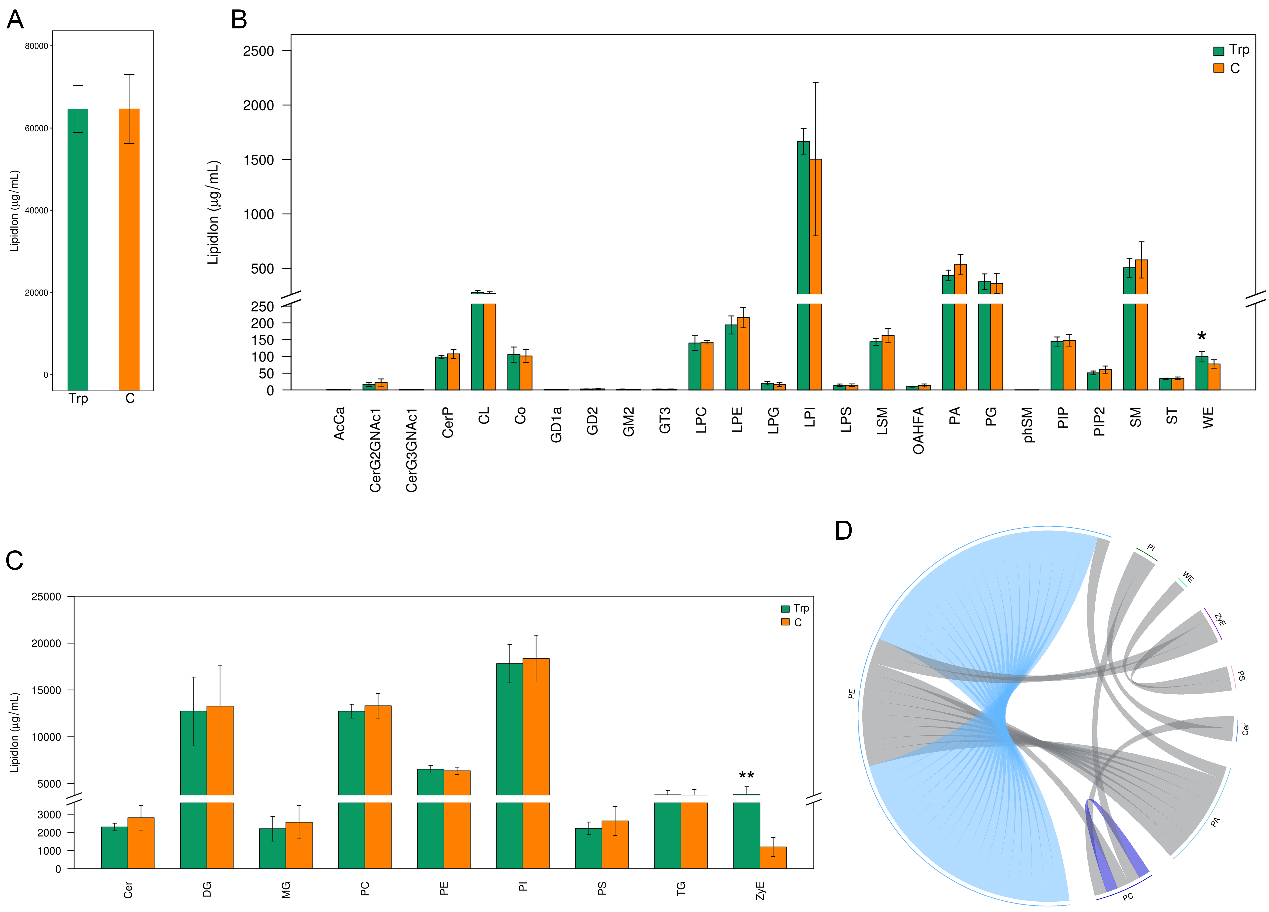


**Figure S6. Liver lipidomics in finishing pigs.** (**A**) Total lipid concentration. (**B**) and (**C**) The content of the various lipid classes. (**D**) Graphical representation of significant correlations among metabolites from different lipid classes or within the same class. Links starting from the inner circle denote significantly different lipid molecules, while arcs in the outer circle represent lipid subclasses. Coloured lines indicate correlations within subclasses, matching the colour of the subclasses. Dark grey lines indicate correlations between subclasses. Values are means ± SEM (n = 5 per group). For statistical analysis, a two-tailed unpaired Student's t-test was used. *P < 0.05, **P < 0.01 vs. the C group
